# Tempo and Mode of Genome Structure Evolution in Insects

**DOI:** 10.3390/genes14020336

**Published:** 2023-01-28

**Authors:** James M. Alfieri, Michelle M. Jonika, Jennifer N. Dulin, Heath Blackmon

**Affiliations:** 1 Department of Biology, Texas A&M University, College Station, TX 77843, USA; 2 Interdisciplinary Program in Ecology and Evolutionary Biology, Texas A&M University, College Station, TX 77843, USA; 3 Interdisciplinary Program in Genetics and Genomics, Texas A&M University, College Station, TX 77843, USA

**Keywords:** genetic drift, chromosome evolution, speciation, karyotype

## Abstract

The division of the genome into discrete chromosomes is a fundamental characteristic of eukaryotic life. Insect taxonomists’ early adoption of cytogenetics has led to an incredible amount of data describing genome structure across insects. In this article, we synthesize data from thousands of species and use biologically realistic models to infer the tempo and mode of chromosome evolution among insect orders. Our results show that orders vary dramatically in the overall rate of chromosome number evolution (a proxy of genome structural stability) and the pattern of evolution (e.g., the balance between fusions and fissions). These findings have important implications for our understanding of likely modes of speciation and offer insight into the most informative clades for future genome sequencing.

## 1. Introduction

Chromosomes are the physical carriers of heritable information from generation to generation. One of the most basic features of a genome is how heritable information is divided into discrete chromosomes and either autosomes or sex chromosomes. Despite their apparent simplicity, these basic features are incredibly variable across the tree of life. Despite a century of work, the development of rules that can explain the varying tempo and mode of chromosomal evolution remains elusive.

Generally, organisms have one crossover per chromosome or chromosome arm per meiosis [1,2]. This pattern leads to one of the most apparent impacts of changes in chromosome number: increases in chromosome number should reduce average linkage among genes, while decreases in chromosome number should increase average linkage among genes. This fact links a rich literature focused on determining the conditions that favor or disfavor recombination to the evolution of gross genome structure [3,4,5,6].

Reductions in chromosome number (i.e., reductions in recombination) can be favored under various conditions that favor the transmission of parental multi-locus genotypes. One example of these multi-locus genotypes is a “coadapted gene complex.” This is a system where multiple polymorphic loci have specific alleles at each locus that, in concert, allow for the production of a high-fitness individual [7,8,9]. Another process that may favor reductions in chromosome numbers is autosomal sexual antagonism. This is a special case of a coadapted gene complex where one gene is the sex-determining locus, and the other gene in the complex is a polymorphic autosomal locus with one allele beneficial to females and another allele beneficial to males. Under sexually antagonistic conditions such as this, an organism will experience segregation load (a reduction in fitness due to random segregation during meiosis). This reduction in fitness can be reduced or eliminated by a fusion between the autosome and a sex chromosome [10,11].

Selection can also favor increases in recombination. As the number of chromosomes increases, so does the number of recombination points. For this reason, an individual with many chromosomes can produce gametes with an increased variety of haplotype combinations than an individual with few chromosomes. This fact was the foundation of a hypothesis that the eusocial Hymenoptera would be more fit if they had a higher rather than lower chromosome number [12]. Early analyses suggested that eusocial Hymenoptera have elevated chromosome numbers. However, later analyses incorporating modern comparative methods illustrated this effect is weak and eusocial Hymenoptera likely maximize genetic diversity through several mechanisms [13]. Sex chromosomes are a classic example of a portion of the genome that evolves reduced recombination. However, even in this case, conditions can favor maintenance or even increases in recombination [14].

Finally, asymmetric meiosis in females offers an additional avenue for selection to drive increases or decreases in chromosome number. Across a broad range of organisms, we know changes in chromosome number can be driven simply by biased transmission of specific chromosome or centromere types to the egg versus polar bodies [15,16,17]. Female meiotic drive explains many cases of rapid remodeling of karyotypes in mammals [18].

As these forces act to change chromosome number, the remodeling of genomes can have dramatic consequences. For example, chromosomal rearrangements can suppress recombination, reducing gene flow and in turn, promoting speciation [19]. Using forward-time population genetic models, Guerrero and Kirkpatrick [20] found fusions can increase in frequency, becoming fixed or remaining polymorphic depending on the population structure and the effect on recombination between locally adapted loci. Guerrero’s and Kirkpatrick’s [20] results suggest chromosome fusions are an important mechanism of local adaptation. Selection can indirectly decrease linkage through chromosome fissions, allowing individual genes to be selected [21]. On the other hand, selection can indirectly increase linkage through chromosome fusions, causing a background selection of newly linked alleles to deleterious alleles [22].

Insects are an ideal group to examine the causes and consequences of chromosomal evolution. Insects are diverse with over one million named species, and are highly variable in chromosome number and in many other traits, such as sex determination systems, population sizes, generation times, habitats, and natural history [23]. Additionally, there is a long history of cytotaxonomy and evolutionary studies leading to vast amounts of data, as well as an abundance of phylogenetic information allowing for model fitting [24].

The tempo and mode of chromosome number evolution remains elusive, but certain questions may help illuminate our knowledge. For the tempo of chromosome number evolution, do different clades evolve at different rates and are these differing rates of evolution responsible for differences in the variation of chromosome number? For the mode of chromosome number evolution, do different clades have different balances between fusions/fissions and do some clades have a significantly higher probability of polyploidy than others? To answer these questions, we modeled chromosome number evolution in eight insect orders to show fundamentally different rates and patterns of chromosome evolution among insect orders. In particular, we found that orders, such as Lepidoptera and Diptera, generally have higher rates of both fusion and fission.

## 2. Materials and Methods

### 2.1. Data Collection

We compiled all available karyotypes from online databases [25,26,27,28,29]. To allow for comparisons across clades, we used the haploid chromosome count of the homogametic sex as a surrogate for the karyotype. For the remainder of the paper, we will refer to this as the chromosome number. For the rate estimates described below, we used the most recent large time-calibrated phylogenies available for each clade: Coleoptera: [30], Lepidoptera: [31], Odonata: [32], Hymenoptera: [13], Hemiptera: [33], Blattodea: [33], Orthoptera: [26], and Diptera: [34].

### 2.2. Inferring Rates of Chromosome Evolution

We used the R package ChromePlus to construct a Markov model of chromosome evolution allowing for three mechanisms of chromosome number change: fusions (δ), fissions (γ), and whole genome duplication (ρ) [18]. Because evidence for whole genome duplication is variable across insects, we additionally fitted a model where the rate of the whole genome duplication was set to zero [35]. Both models were fit in a Bayesian framework using R package diversitree [36]. In cases where the available phylogeny included a posterior distribution of possible phylogenies, our models were fit on each of 100 randomly selected phylogenies. Each Markov Chain Monte Carlo (MCMC) run was initialized with parameter values drawn from a uniform distribution from 0 to 1. We applied an exponential prior with a shape parameter of 2 to avoid sampling unrealistically high rates. All MCMCs were run for 1000 generations. While most MCMCs converged quickly (e.g., 20–30 generations), a handful took much longer (e.g., 400–500 generations). Therefore, to allow for a consistent approach across datasets, we discarded the first 700 generations of all MCMCs as burnin. For clades with a single tree, all 300 post-burnin samples were retained as the posterior distribution. For datasets with a posterior distribution of trees, we kept 3 samples from each of the 100 trees to yield a posterior distribution of 300. All reported rates are lambda parameters for exponential distributions describing the expected waiting time for a transition to occur and are in units of millions of years. One challenge to comparing across clades is a scaling of rate estimates caused by errors in the depth of phylogenies. For instance, if a phylogeny is stretched to twice its “true” depth, rate estimates will be one-half of the “true” value. For this reason, we also evaluated the normalized rates ratio—calculated as the rate of fusion divided by the sum of the rate of fusion and fission. The normalized rates ratio is not impacted by tree depth and allows for comparisons independent of errors in tree depth.

Scripts for all analyses are available in a GitHub repository (https://github.com/coleoguy/strucstability accessed on 4 January 2023).

## 3. Results

### 3.1. Data Collection

We downloaded 16,795 records of insect karyotypes. This data included records for 28 orders of insects and over 4000 different genera of insects. Coleoptera had the most reported karyotypes with 4735, while several orders were represented by only single records (i.e., Grylloblattodea, Mantophasmatodea, and Zoraptera). For comparative analyses we limited our model fitting to orders that had at least 150 available karyotypes: Blattodea, Coleoptera, Diptera, Hemiptera, Hymenoptera, Lepidoptera, Odonata, and Orthoptera (Table 1).

**Figure 1:**
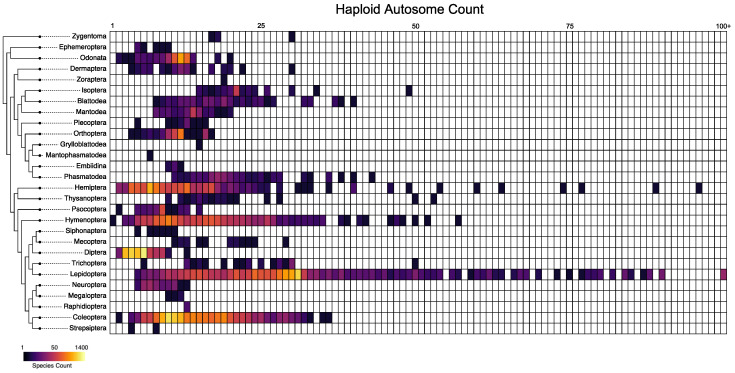
Haploid autosome counts in insects. Each row represents an order of insects and each column indicates a haploid autosome count for the homogametic sex. The shading of each cell represents the number of records with a given haploid count with colors on a log scale. The final column holds the count of all haploid autosome numbers of 100 or higher (22 all in the order, Lepidoptera).

Across our entire dataset, chromosome number ranged from one in the ant, *Myrmecia croslandi*, to 223 in the butterfly, *Polyommatus atlantica* (Figure 1). The most common haploid chromosome count was six with 1612 records (1371 of which were from Diptera). However, several other counts were nearly as common. Chromosome counts of 10, 11, and 12 were each reported in over 1000 species. Lepidoptera had the highest variance of chromosome count (171.5), while Diptera had the lowest variance (1.66).

### 3.2. Rate Estimates

**Figure 2:**
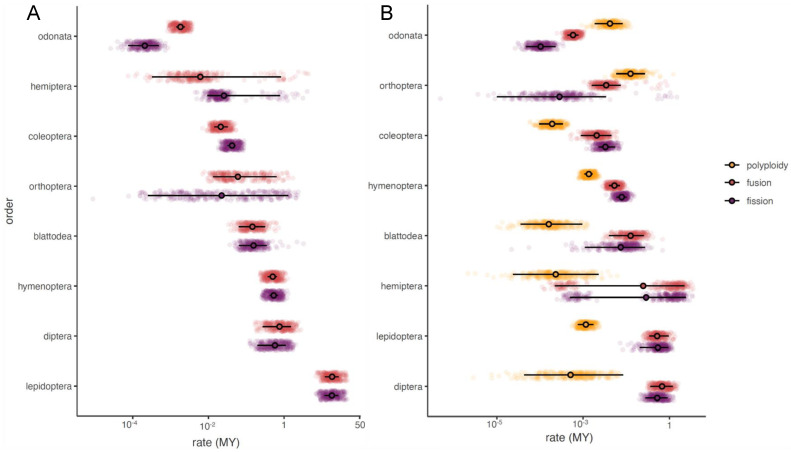
Rates of chromosome evolution in insect orders. In each plot, we show the distribution of the estimated rates for each order of insects included in the study. Rates of chromosome evolution are plotted on a log scale, but unlogged values are shown at the tick marks for ease of interpretation. The credible interval for each statistic is shown as a black line while the mean is indicated with a black circle. (**A**) simple model with fissions and fusions. (**B**) complex model with fission, fusions, and polyploidy. The insect clades are ordered by mean rate in chromosome evolution.

**Figure 3:**
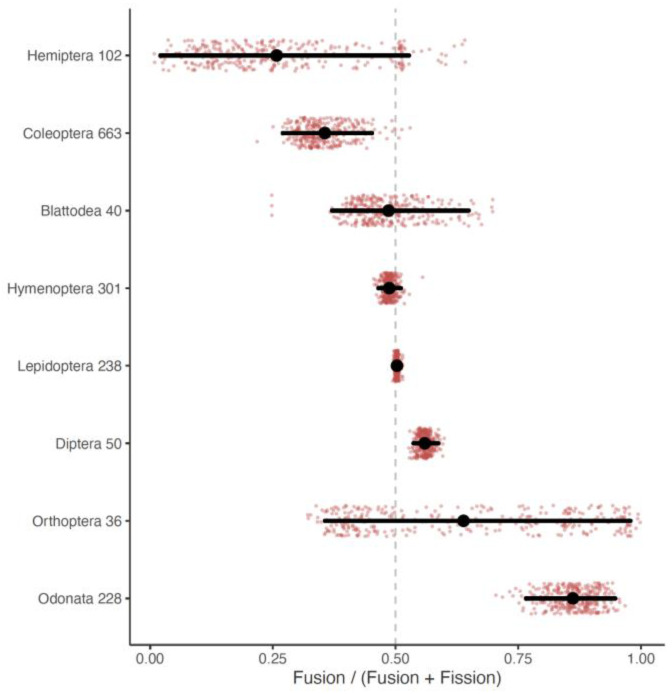
Normalized rates ratio for chromosome evolution. Each row of the plot represents an order; the number after the order name indicates the number of tips included in the analysis. The horizontal axis indicates the normalized rates ratio. The black line indicates the credible interval for the rates ratio and the mean is indicated by the larger black circle.

In Blattodea, we had a total of 40 species present in our phylogeny and karyotype dataset. The species in our karyotype dataset had a haploid chromosome count range from 8 to 49, while the 40 species used in our analysis had a haploid chromosome count range from 9 to 49. Using the simple two-parameter model, we estimated rates of 0.201 (95% HPD Interval: 0.09–0.36) and 0.223 (95% HPD Interval: 0.08–0.37) for fusions and fissions, respectively (Figure 2). Using the more complex model, we estimated rates of 0.119 (95% HPD Interval: 0.02–0.21), 0.082 (95% HPD Interval: 0–0.18), and 0.001 (95% HPD Interval: 0–0.004) for fusion, fission, and polyploidy, respectively. We calculated the mean normalized rates ratio as 0.49 (95% HPD Interval: 0.37–0.65) (Figure 3).

In Coleoptera, we had a total of 663 species present on our phylogeny and in our karyotype dataset. The species in our karyotype dataset had a haploid chromosome count range from 2 to 36, while the 663 species used in our analysis had a haploid chromosome count range from 4 to 36. Using the simple two-parameter model, we estimated rates of 0.036 (95% HPD Interval: 0.02–0.05) and 0.064 (95% HPD Interval: 0.05–0.08) for fusions and fissions, respectively (Figure 2). Using the more complex model, we estimated rates of 0.016 (95% HPD Interval: 0.003–0.03), 0.025 (95% HPD Interval: 0.015–0.04), and 0.001 (95% HPD Interval: 0.0004–0.002) for fusion, fission, and polyploidy, respectively. We calculated the mean normalized rates ratio as 0.36 (95% HPD Interval: 0.27–0.45) (Figure 3).

In Diptera, we had a total of 50 species present on our phylogeny and in our karyotype dataset. The species in both our karyotype dataset and the species used in the analysis had a haploid chromosome count range from 2 to 13. The tips represented on our tree include 50 of 51 families for which karyotype data is available. Using the simple two-parameter model, we estimated rates of 0.863 (95% HPD Interval: 0.30–1.38) and 0.678 (95% HPD Interval: 0.24–1.10) for fusions and fissions, respectively (Figure 2). Using the more complex model, we estimated rates of 0.692 (95% HPD Interval: 0.33–1.2), 0.521 (95% HPD Interval: 0.25–0.89), and 0.008 (95% HPD Interval: 0–0.03) for fusion, fission, and polyploidy, respectively. We calculated the mean normalized rates ratio as 0.56 (95% HPD Interval: 0.54–0.59) (Figure 3).

In Hemiptera, we had a total of 102 genera present on our phylogeny and in our karyotype dataset. The species in our karyotype dataset had a haploid chromosome count range from 2 to 96, while the 102 species used in our analysis had a haploid chromosome count range from 2 to 25. Using the simple two-parameter model, we estimated rates of 0.106 (95% HPD Interval: 0–0.79) and 0.121 (95% HPD Interval: 0.01–0.74) for fusions and fissions, respectively (Figure 2). Using the more complex model, we estimated rates of 0.984 (95% HPD Interval: 0–2.23), 0.927 (95% HPD Interval: 0–2.13), and 0.003 (95% HPD Interval: 0–0.01) for fusion, fission, and polyploidy, respectively. We calculated the mean normalized rates ratio as 0.26 (95% HPD Interval: 0.02–0.53) (Figure 3).

In Hymenoptera, we had a total of 301 species present on our phylogeny and in our karyotype dataset. The species that could be included in the comparative analysis (species that were shared between the phylogeny and karyotype dataset) had a haploid chromosome count from 1 to 57. The species in both our karyotype dataset and the species used in the analysis had a haploid chromosome count range from 1 to 29. Using the simple two-parameter model, we estimated rates of 0.555 (95% HPD Interval: 0.42–0.69) and 0.583 (95% HPD Interval: 0.46–0.73) for fusions and fissions, respectively (Figure 2). Using the more complex model, we estimated rates of 0.042 (95% HPD Interval: 0.03–0.06), 0.064 (95% HPD Interval: 0.04–0.08), and 0.009 (95% HPD Interval: 0–0.01) for fusion, fission, and polyploidy, respectively. We calculated the mean normalized rates ratio as 0.49 (95% HPD Interval: 0.47–0.51) (Figure 3).

In Lepidoptera, we had a total of 238 species present on our phylogeny and in our karyotype dataset. The species in our karyotype dataset had a haploid chromosome count range from 5 to 223, while the 238 species used in our analysis had a haploid chromosome count range from 8 to 141. Using the simple two-parameter model, we estimated rates of 13.005 (95% HPD Interval: 8.24–17.42) and 12.842 (95% HPD Interval: 8.30–17.26) for fusions and fissions, respectively (Figure 2). Using the more complex model, we estimated rates of 0.508 (95% HPD Interval: 0.25–0.79), 0.548 (95% HPD Interval: 0.15–0.81), and 0.008 (95% HPD Interval: 0–0.01) for fusion, fission, and polyploidy, respectively. We calculated the mean normalized rates ratio as 0.50 (95% HPD Interval: 0.49–0.51) (Figure 3).

In Odonata, we had a total of 203 species present on our phylogeny and in our karyotype dataset. The species in our karyotype dataset had a haploid chromosome count range from 2 to 20. The species that could be included in the comparative analysis (species that were shared between the phylogeny and karyotype dataset) had a haploid chromosome count from 4 to 14. Using the simple two-parameter model, we estimated rates of 0.004 (95% HPD Interval: 0–0.01) and 0.001 (95% HPD Interval: 0–0.001) for fusions and fissions, respectively (Figure 2). Using the more complex model we estimated rates of 0.004 (95% HPD Interval: 0–0.005), 0.001 (95% HPD Interval: 0–0.001), and 0.035 (95% HPD Interval: 0.01–0.06) for fusion, fission, and polyploidy, respectively. We calculated the mean normalized rates ratio as 0.86 (95% HPD Interval: 0.77–0.95) (Figure 3).

In Orthoptera, we had a total of 36 species present on our phylogeny and in our karyotype dataset. The species in our karyotype dataset had a haploid chromosome count range from 4 to 17, while the 36 species used in our analysis had a haploid chromosome count range from 4 to 12. Using the simple two-parameter model, we estimated rates of 0.161 (95% HPD Interval: 0.02–0.64) and 0.216 (95% HPD Interval: 0–1.05) for fusions and fissions, respectively (Figure 2). Using the more complex model, we estimated rates of 0.039 (95% HPD Interval: 0.01–0.06), 0.025 (95% HPD Interval: 0–0.01), and 0.121 (95% HPD Interval: 0.04–0.22) for fusion, fission, and polyploidy, respectively. We calculated the mean normalized rates ratio as 0.64 (95% HPD Interval: 0.36–0.98) (Figure 3).

## 4. Discussion

Our study shows that insect orders have fundamentally different rates and patterns of chromosome evolution. In particular, we find that some orders, such as Lepidoptera and Diptera, have generally higher rates of both fusions and fissions. The high rates of chromosome evolution that we document in Lepidoptera is consistent with previous analyses that used far fewer species than the current study [37]. More broadly, Lepidoptera have long been assumed to have generally high rates of chromosome evolution based on the broad range of haploid chromosome counts present in the clade (Figure 1) [38].

In contrast, our finding of high rates of chromosome evolution in Diptera is more surprising. Diptera has long been recognized as one of the least variable insect orders with regard to chromosome count [28,39]. It has been suggested that this is due to an inability to generate new chromosome ends beyond those present in the six basic Muller elements (possibly due to a lack of telomerase [40,41]. Our estimate of the normalized rates ratio is also consistent with some limits on increases in chromosome number. The credible interval of our estimate was entirely greater than 0.5, suggesting that fusion rates are constantly higher than fission rates. However, our data collection shows that a total of 133 (~4%) Diptera records are for species with seven or more chromosomes. These 133 cases are also not taxonomically clustered; in fact, 15 of the 51 families for which we have data have at least one species with more than six chromosomes. Among these 15 families, three stand out as having a large proportion of the records. The family, Tabanidae, has 73 records, 34 of which are species with greater than six chromosomes. This family includes the species, *Haematopota subcylindrica*, which has the highest chromosome number of any Diptera with 13 chromosomes. Similarly, the families, Stratiomyidae and Bombyliidae, also contain significant numbers of species with greater than six chromosomes (24 and 29 species, respectively). This fact combined with the generally high rates of fusions and fissions suggest that as a whole, Diptera may have more dynamic genomes than previous work has suggested. Future studies would benefit from an analysis of rates and patterns of chromosome evolution within these families and contrasts between these families and more typical families (e.g., Drosophilidae and Culicidae) that lack high chromosome number species.

Similar to Diptera, Odonata showed a bias towards more fusions than fissions (rates ratio was greater than 0.5). Unlike in Diptera, there is no indication that constraints are present due to a loss of telomerase. One possible explanation for the pattern observed in Odonata is the presence of frequent sexual antagonism. Ancestrally, Odonata is thought to have possessed an XO sex determination system [29]. This system is found in approximately 90% (659 of 706) of all records for the order. The remaining species have fusions of autosomes and sex chromosomes that lead to XY and XXY sex chromosome systems. It may be that these fusions are favored as a route to resolve sexual antagonism present on autosomes [10]. Future work should test for an excess of these fusions in Odonata [11].

Coleoptera was the only clade that had a credible interval for the normalized rates ratio that fell completely under 0.5 (Figure 3). The rates ratios as depicted in Figure 3 are based on the simple model that only includes fission and fusion. One possible explanation then could be that some species included in the Coleoptera analysis have experienced whole genome duplications and that the absence of this from the model is leading to an overestimate of the fission rate. In fact, previous work has suggested that whole or large scale genome duplications may have occurred early in some Coleoptera lineages [35]. To test whether the absence of polyploidy in our model led to a bias in our rates ratio, we recalculated the normalized rates ratio for Coleoptera based on the fusion and fission rates estimated under the complex model. Using this approach, we calculated a rates ratio of 0.38 (credible interval 0.12–0.56), 87% of samples are below 0.5 (indicating higher fission than fusion rates). We interpreted this as an indication that Coleoptera have at least a small bias towards fissions over fusions.

One potential concern in comparative analyses of this nature is that a single species or small number of species may exhibit such a strong signal (e.g., high rates of chromosome number evolution) that it overrides any countervailing signal (e.g., low rates of chromosome number evolution) from the rest of the phylogeny. This has been shown to be a weak point in some phylogenetic comparative methods [42,43]. To test whether this might drive the more extreme rate estimates, we evaluated the sensitivity of our rate estimates to the presence or absence of clades with particularly extreme chromosome counts. For this evaluation, we focused on Coleoptera (a clade with moderate rates) and Lepidoptera (a clade with high rates). In the Lepidoptera dataset, we found that the genus, *Polyommatus*, had both the highest haploid chromosome number and the highest variance in haploid chromosome number. To test for the impact of this genus on our overall rate estimates in Lepidoptera, we removed the 21 *Polyommatus* species from our analysis and repeated the rate estimate as described above. For Coleoptera, we followed a similar procedure; we identified the genus, *Calathus*, as a clade with high variance in haploid chromosome count and more than 10 species, and removed it before repeating our rate estimates. For both orders, we fit a simple model of chromosome evolution with only fusions and fissions. This sensitivity analysis showed that as expected, these highly variable clades (i.e., *Polyommatus* and *Calathus*) did drive higher rates. However, the difference between clades was striking. In Coleoptera, rates reduced by approximately 5% with the removal of the *Calathus* species from the analysis (Supplementary Materials Figure S1). In contrast, the removal of the *Polyommatus* species from the analysis of Lepidoptera led to approximately a 50% reduction in the rates estimated for Lepidoptera (Supplementary Materials Figure S2). While this is a dramatic shift, we note that the rate estimates are still higher than Diptera (the next fastest evolving order).

While the focus of our analyses is on the evolution of chromosome number as a proxy for genome structural stability, sex chromosomes also offer insight into the labile nature of insect genomes. In particular, intersecting data on sex chromosome systems and the number of autosomes offers some insights into the frequent and unique role that fusions often play in the remodeling of karyotypes and sex chromosome systems. The databases that we used for collecting chromosome number data also report sex chromosome systems. In some cases, this data is present for almost all species and is quite variable. For instance, in the order of Coleoptera, sex chromosome systems can be clustered into four large groups: 666 XO species, 2898 XY species, 177 NeoXY species, and 186 species, with complex sex chromosome systems (e.g., XXY, XYY, etc.). This variation is broadly spread across subgroups. One hundred thirty-four genera contain species with differing sex chromosome systems (e.g., XX/XY species and XX/XO species).

Fusions often drive transitions between sex chromosome systems. For instance, transitions from XO to XY and transitions from XY to NeoXY systems are typically the result of an autosome fusion with an ancestral sex chromosome. We see strong support for this type of genome rearrangement in our data. Specifically, we find that within 43% (25 of 58) of genera with both XO and XY species, the mean number of autosomes in XY species is lower than the mean number of autosomes in XO species (suggesting the transition was driven by an autosome fusing to the ancestral X). We find an even more striking pattern in those genera that have both XY and NeoXY species. In 70% of these genera (33 of 47), the NeoXY species have a mean number of autosomes lower than the XY species.

This pattern is not limited to Coleoptera. We document a similar pattern in the distribution of autosome numbers and sex chromosome systems in Odonata. Odonata is an ancestrally XX/XO order, and this continues to be the most common system observed (659 species). However, 39 species have been reported with XX/XY sex chromosome systems. Seventeen genera contain XX/XO and XX/XY species, suggesting the XX/XY species are relatively recently evolved. In Odonata, 88% of the genera (15 of 17) have a mean number of autosomes in the XX/XY species that is lower than the mean number of autosomes in the XX/XO species.

Our study illustrates that both the tempo and mode of chromosome evolution is strikingly variable across insect orders. With regard to tempo, we find that some orders (e.g., Lepidoptera and Diptera) have rates that are considerably higher than rates in other orders of insects. This suggests that in these clades in particular, mechanisms of speciation that are dependent on or accelerated in the presence of structural changes in the genome may be more important [44,45,46,47]. In contrast, other orders such as Odonata, appear to have genomes that are structurally more stable. This suggests that in this clade speciation under models that depend on structural changes will be less frequent. Finally, our study and the approaches that we use provide important insights with regard to future sequencing projects. For instance, our analyses in Diptera point to the need for more whole-genome sequencing in clades such as Tabanidae, Stratiomyidae, and Bombyliidae to understand how species in these group are able to form new chromosomes while species in other clades appear unable to form new chromosomes.

## Figures and Tables

**Table 1:** Rate estimates for included insect orders.

	2-Parameter Model		3-Parameter Model		
	Fusion	Fission	Fusion	Fission	Polyploidy
Blattodea	0.201	0.223	0.119	0.082	0.001
Coleoptera	0.036	0.064	0.016	0.025	0.001
Diptera	0.863	0.678	0.692	0.521	0.008
Hemiptera	0.106	0.121	0.984	0.927	0.003
Hymenoptera	0.555	0.583	0.042	0.064	0.009
Lepidoptera	13.005	12.842	0.508	0.548	0.008
Odonata	0.004	0.001	0.004	0.001	0.035
Orthoptera	0.161	0.216	0.039	0.025	0.121

## Data Availability

Raw data and scripts for all analyses are available in a GitHub repository (https://github.com/coleoguy/strucstability accessed on 4 January 2023).

## Supplementary Materials

The following supporting information can be downloaded at: https://www.mdpi.com/article/10.3390/genes14020336/s1, Figure S1: Sensitivity analysis of Lepidoptera; Figure S2: Sensitivity analysis of Coleoptera.
