# Supplementary figures and images for: Tempo and Mode of Genome Structure Evolution in Insects
Source: Genes (Basel). 2023 Jan 28;14(2):336. doi: 10.3390/genes14020336 (PMC9957073; doi:10.3390/genes14020336)

Figure S1. Sensitivity analysis of Lepidoptera

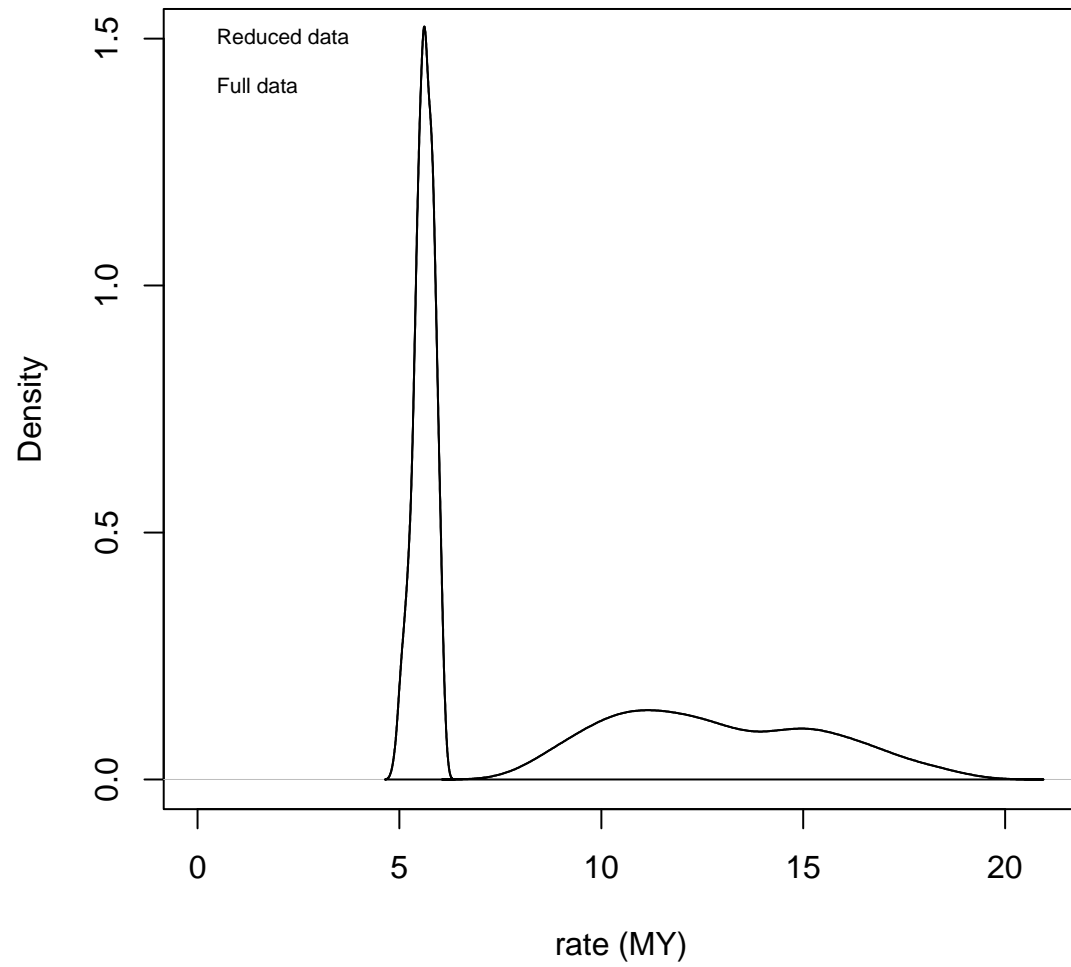

Figure S2. Sensitivity analysis of Coleoptera

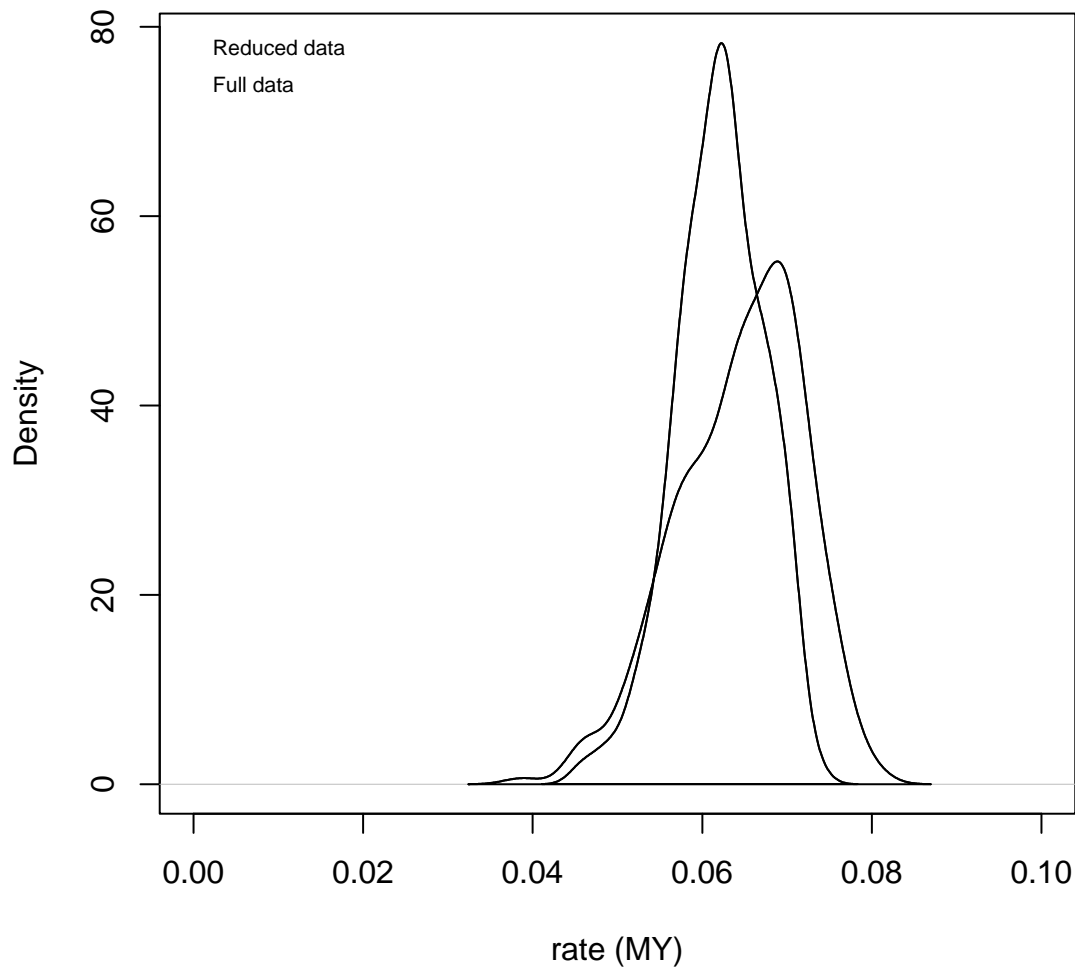

Supplement: Supplementary file 1 [file genes-14-00336-s001.zip › genes-2178083-supplementary.pdf]
